# Single-Channel EEG Features Reveal an Association With Cognitive Decline in Seniors Performing Auditory Cognitive Assessment

**DOI:** 10.3389/fnagi.2022.773692

**Published:** 2022-05-30

**Authors:** Lior Molcho, Neta B. Maimon, Noa Regev-Plotnik, Sarit Rabinowicz, Nathan Intrator, Ady Sasson

**Affiliations:** ^1^Neurosteer Inc., New York, NY, United States; ^2^The School of Psychological Sciences, Tel Aviv University, Tel Aviv, Israel; ^3^Dorot Geriatric Medical Center, Netanya, Israel; ^4^Blavatnik School of Computer Science, Tel Aviv University, Tel Aviv, Israel; ^5^Sagol School of Neuroscience, Tel Aviv University, Tel Aviv, Israel

**Keywords:** EEG (electroencephalography), brain activity, cognitive decline, cognitive impairment, cognitive assessment, mini-mental state examination (MMSE), machine learning (ML), wavelet packet

## Abstract

**Background:**

Cognitive decline remains highly underdiagnosed despite efforts to find novel cognitive biomarkers. Electroencephalography (EEG) features based on machine-learning (ML) may offer a non-invasive, low-cost approach for identifying cognitive decline. However, most studies use cumbersome multi-electrode systems. This study aims to evaluate the ability to assess cognitive states using machine learning (ML)-based EEG features extracted from a single-channel EEG with an auditory cognitive assessment.

**Methods:**

This study included data collected from senior participants in different cognitive states (60) and healthy controls (22), performing an auditory cognitive assessment while being recorded with a single-channel EEG. Mini-Mental State Examination (MMSE) scores were used to designate groups, with cutoff scores of 24 and 27. EEG data processing included wavelet-packet decomposition and ML to extract EEG features. Data analysis included Pearson correlations and generalized linear mixed-models on several EEG variables: Delta and Theta frequency-bands and three ML-based EEG features: VC9, ST4, and A0, previously extracted from a different dataset and showed association with cognitive load.

**Results:**

MMSE scores significantly correlated with reaction times and EEG features A0 and ST4. The features also showed significant separation between study groups: A0 separated between the MMSE < 24 and MMSE ≥ 28 groups, in addition to separating between young participants and senior groups. ST4 differentiated between the MMSE < 24 group and all other groups (MMSE 24–27, MMSE ≥ 28 and healthy young groups), showing sensitivity to subtle changes in cognitive states. EEG features Theta, Delta, A0, and VC9 showed increased activity with higher cognitive load levels, present only in the healthy young group, indicating different activity patterns between young and senior participants in different cognitive states. Consisted with previous reports, this association was most prominent for VC9 which significantly separated between all level of cognitive load.

**Discussion:**

This study successfully demonstrated the ability to assess cognitive states with an easy-to-use single-channel EEG using an auditory cognitive assessment. The short set-up time and novel ML features enable objective and easy assessment of cognitive states. Future studies should explore the potential usefulness of this tool for characterizing changes in EEG patterns of cognitive decline over time, for detection of cognitive decline on a large scale in every clinic to potentially allow early intervention.

**Trial Registration:**

NIH Clinical Trials Registry [https://clinicaltrials.gov/ct2/show/results/NCT04386902], identifier [NCT04386902]; Israeli Ministry of Health registry [https://my.health.gov.il/CliniTrials/Pages/MOH_2019-10-07_007352.aspx], identifier [007352].

## Introduction

Cognitive decline is characterized by impairments in various cognitive functions such as memory, orientation, language, and executive functions, expressed more than is anticipated for an individual’s age and education level (Plassman et al., 2010). Cognitive decline with memory deficit indications is associated with a high-risk for developing dementia and Alzheimer’s disease (AD) (Ritchie and Touchon, 2000). Dementia is recognized as one of the most significant medical challenges of the future. So much so, that it has already reached epidemic proportions, with prevalence roughly doubling every 5 years in populations over the age of 65 (van der Flier and Scheltens, 2005). This rate is expected to increase unless therapeutic approaches are found to prevent or stop disease progression (Hebert et al., 2013). Since AD is the most prevalent form of dementia, responsible for about 60–70% of cases (Qiu et al., 2009), it remains the focus of clinical trials. To date, most clinical trials that include a disease-modifying treatment fail to demonstrate clinical benefits in symptomatic AD patients. This could be explained by the late intervention that occurs after neuropathological processes have already resulted in substantial brain damage (Galimberti and Scarpini, 2011). Hence, the discovery of predictive biomarkers for preclinical or early clinical stages, such as cognitive decline, is imperative (Jack et al., 2011). Cognitive decline may be detected several years before dementia onset with known validated tools (Hadjichrysanthou et al., 2020). Interventions starting early in the disease process, before substantial neurodegeneration has taken place, can change the progression of the disease dramatically (Silverberg et al., 2011). Yet, there is still no universally recommended screening tool that satisfies all needs for early detection of cognitive decline (Cordell et al., 2013).

The most commonly used screening tool for cognitive assessment in the elderly population is the Mini-Mental State Examination (MMSE) (Folstein et al., 1975). The MMSE evaluates cognitive function, with a total possible score of 30 points. Patients who score below 24 would typically be suspected of cognitive decline or early dementia (Tombaugh and McIntyre, 1992). However, several studies have shown that sociocultural variables, age, and education, as well as tester bias, could affect individual scores (Brayne and Beardsall, 1990; Crum et al., 1993; Shiroky et al., 2007). Furthermore, studies report a short-term practice effect for subjects in AD trials and diagnostic studies resulting from repeated exposure to the MMSE (Chapman et al., 2016).

Objective cognitive assessment based on brain activity measurements would be preferable to subjective clinical evaluations using pen-and-paper assessment tools like the MMSE. However, such objective methods are often cumbersome and expensive. Electroencephalography (EEG) offers a non-invasive and relatively inexpensive screening tool for cognitive assessment (Cassani et al., 2018). Frontal asymmetry among the activity in the left and right hemispheres, peak frequency in resting-state EEG, and response time in sensory ERP are found to correlate with MMSE scores (Doan et al., 2021). EEG studies investigating cognitive decline highlight the role of Theta power as a possible indicator for early detection of cognitive decline (Missonnier et al., 2007; Deiber et al., 2015). For example, it was found that frontal Theta activity differs substantially in cognitively impaired subjects performing cognitive tasks, compared to healthy seniors. A lack of increased Theta activity was shown to serve as a predictor of cognitive decline progression (Deiber et al., 2009). When performing tasks involving working memory, frontal Theta activity increases with the expected increase of the cognitive load levels (Jensen and Tesche, 2002). Working memory manipulation is one of the ways to modify cognitive load, as explained by the Cognitive Load Theory (CLT). Since working memory capacity is limited, performing a higher difficult task results in simultaneous processing of information elements, which leads to higher cognitive load (Sweller, 2011). Studies using EEG repeatedly show frontal Theta increase with higher cognitive load and task difficulty (Antonenko et al., 2010). Studies examining resting-state EEG found that Alpha-to-Theta ratio decreased as the MMSE scores decreased (Choi et al., 2019). A recent study suggest that novel diagnostic classification based on EEG signals could be even more useful than frontal Theta for differentiating between clinical stages (Farina et al., 2020).

The development of machine learning (ML), alongside advancement in signal processing, has largely contributed to the extraction of useful information from the raw EEG signal (Dauwels et al., 2010a). Novel techniques are capable of exploiting the large amount of information on time-frequency processes in a single recording (Pritchard et al., 1994; Babiloni et al., 2004). Recent studies demonstrated novel measures of EEG signals for identification of cognitive impairment with high accuracy, using classifiers based on neural networks, wavelets, and principal component analysis (PCA), indicating the relevance of such methods for cognitive assessment (Cichocki et al., 2005; Melissant et al., 2005; Lehmann et al., 2007; Ahmadlou et al., 2010; Meghdadi et al., 2021).

However, most studies in this field have several constraints. Most commonly, such studies use multichannel EEG systems to characterize cognitive decline. The difficulty with multichannel EEG is the long setup time, the requirement of specially trained technicians, as well as the need for professional interpretation of the results. This makes the systems costly and not portable, thus not suitable for wide-range screening in community clinics. Consequently, these systems are not included in the usual clinical protocol for cognitive decline detection. This emphasizes the need for additional cost-effective tools with easy setup and short assessment times, to possibly allow earlier detection of cognitive decline in the community.

A recent study (Khatun et al., 2019) examined differences in responses to auditory stimuli between cognitively impaired and healthy subjects and concluded that cognitive decline can be characterized using data from a single EEG channel. Specifically, using data from frontal electrodes, the authors extracted features that were later used in classification models to identify subjects with cognitive impairments. Additional studies (Choi et al., 2019; Doan et al., 2021) found prefrontal EEG effective for screening dementia and, specifically, frontal asymmetry as a potential EEG variable for dementia detection. These results contribute to the notion that a prefrontal single-channel EEG can be used as an efficient and convenient way for assessing cognitive decline. However, the risk of overfitting the data in such classification studies should be addressed to ensure generalization capabilities, especially with a small sample size. Studies that use the same dataset for training as well as feature extraction (Kashefpoor et al., 2016; Cassani et al., 2017; Khatun et al., 2019) extend the risk of overfitting the data. For generalization of the data, the features should be examined in different datasets and be made to provide consistency in the results of new datasets. Furthermore, measuring the correlations of the extracted features with standard clinical measurements (like the MMSE score) or behavioral results of cognitive tasks [like reaction times (RTs) and accuracy] may be highly valuable for validation of novel EEG features.

In this study, we evaluated the ability of an easy-to-use single-channel EEG system to potentially detect cognitive decline in an elderly population. The EEG signal was decomposed using mathematical models of harmonic analysis, and machine-learning (ML) methods were used to extract EEG features. The pre-extracted EEG features used in this study were validated in previous studies performed on young healthy subjects (Maimon et al., 2020, 2021, 2022; Bolton et al., 2021). A short auditory cognitive assessment utilizing auditory stimuli was used. The auditory cognitive assessment included a simple auditory detection task with two difficulty levels (low and high), and a resting-state task. Previous findings show that recording EEG during active engagement in cognitive and auditory tasks offers distinct features and may lead to better discrimination power of brain states (Ghorbanian et al., 2013). Furthermore, using auditory stimulation detection is linked directly to attentional processes of the working memory system and can be used to manipulate WM load, as shown by EEG studies (Berti and Schröger, 2001; Lv et al., 2010). To continue this notion, we used an auditory assessment battery with musical stimuli. It was previously shown that musical stimuli elicit stronger activity than using visual cues such as digits and characters (Tervaniemi et al., 1999).

This pilot study aims to evaluate the ability of a frontal single-channel EEG system to assess cognitive decline in an elderly population, recognizing the importance of providing an accurate, low-cost alternative for cognitive decline assessment. Several hypotheses were formed in this study: (1) The EEG features activity will correlate to the MMSE scores (in the senior groups); (2) The EEG features that show correlation to the MMSE scores will also differentiate between the lower MMSE groups and the healthy young group; (3) Some of the EEG features will correlate to cognitive load (elicited by the tasks); and (4) This pattern of activity, which refers to the difficulty of the task, could be absent in patients with low MMSE scores.

## Materials and Methods

### Participants

#### Senior Participants

Ethical approval for this study was granted by the Ethics Committee (EC) of Dorot Geriatric Medical Center on July 01, 2019. Israeli Ministry of Health (MOH) registry number MOH_2019-10-07_007352. NIH Clinical Trials Registry number NCT04386902, URL: https://clinicaltrials.gov/ct2/show/NCT04386902.

Sixty patients from the inpatient rehabilitation department at Dorot Geriatric Medical Center were recruited for this study. For the full demographic details, see Table 1. The overall mean age was 77.55 (9.67) years old. There was a wide range of ages for each group, with no significant age difference between the groups. Participant groups consisted of 47% females and 53% males. Among the patients, 82% were hospitalized for orthopedic rehabilitation, and 18% due to various other causes. Among the patients who had surgery, an average of 27 (16.3) days had passed since the surgery. Potential subjects were identified by the clinical staff during their admissions to the inpatient rehabilitation department. All subjects were hospitalized at the center and were chosen based on inclusion criteria specified in the study protocol. The patients underwent a MMSE by an occupational therapist upon hospital admission, and this score was used to screen patients who had scores between 10 and 30. All subjects were also evaluated for their abilities to hear, read, and understand instructions for the discussion of Informed Consent Form (ICF), as well as for the auditory task. Patients that spoke English, Hebrew, and Russian were provided with the appropriate ICF and auditory task in the language they could read and understand. All participants provided ICF according to the guidelines outlined in the Declaration of Helsinki. Patients that showed any verbal or non-verbal form of objection were not included in the study. Other exclusion criteria included MMSE score lower than 10; the presence of several neurological comorbidities (intended to exclude patients with other neurological conditions that could affect the results); damage to the integrity of the scalp and/or skull, and skin irritation in the facial and forehead area; significant hearing impairments; and a history of drug abuse.

**TABLE 1:** Demographic information of the senior groups included in the analysis.

	Groups	MMSE ≥ 28	MMSE 24–27	MMSE < 24
Total	MMSE scores	28–30	24–27	17–23
	*n*	17	16	17
	MMSE	28.88 (0.78)	25.64 (0.66)	20.46 (2.06)
	Age	74.77 (8.05)	75.42 (7.36)	79.26 (8.57)
	Age *t*-tests	MMSE ≥ 28 vs. MMSE 24–27, *t* = –0.43, *p* = 0.67	MMSE ≥ 28 vs. MMSE < 24, *t* = –1.62, *p* = 0.115	MMSE 24–27 vs. MMSE < 24, *t* = –1.73, *p* = 0.25
Male	MMSE scores	28–30	24–27	17–23
	*n*	5	7	11
	MMSE	28.4 (0.55)	25.86 (0.69)	21.1 (1.92)
	Age	74.4 (8.55)	73.86 (5.55)	78.27 (7.16)
	Age *t*-tests	MMSE ≥ 28 vs. MMSE 24–27, *t* = 0.11, *p* = 0.91	MMSE ≥ 28 vs. MMSE < 24, *t* = –0.81, *p* = 0.45	MMSE 24–27 vs. MMSE < 24, *t* = –1.47, *p* = 0.16
Female	MMSE scores	28–30	24–27	17–23
	*n*	12	9	6
	MMSE	29.08 (0.9)	25.56 (1.01)	20.17 (2.48)
	Age	74.83 (4.99)	77.11 (7.75)	79.67 (10.48)
	Age *t*-tests	MMSE ≥ 28 vs. MMSE 24–27, *t* = 0.77, *p* = 0.46	MMSE ≥ 28 vs. MMSE < 24, *t* = –1.07, *p* = 0.325	MMSE 24–27 vs. MMSE < 24, *t* = –0.51, *p* = 0.622
MMSE males vs. females		*t* = –1.91, *p* = 0.08	*t* = 0.71, *p* = 0.49	*t* = 0.79, *p* = 0.45
Age males vs. females		*t* = –0.1, *p* = 0.92	*t* = –0.98, *p* = 0.345	*t* = –0.29, *p* = 0.78

*Averages are shown for total (all participants), and for males and females separately. t and p values of the comparisons between mean ages of the MMSE groups are presented for total and for males and females separately. Additionally, t and p-values of the comparisons of age and MMSE between the genders are presented in the last rows.*

In total, 50 of the 60 recruited patients completed the auditory task, and their EEG data was used. Ten patients signed the ICF and were included in the overall patient count but were excluded from data analysis due to their desire to stop the study, or because of technical problems during the recording.

#### Healthy Young Participants

Twenty-two healthy students participated in this study for course credit. The overall mean age was 24.09 (2.79) years old. Participant group consisted of 60% females and 40% males. Ethical approval for this study was granted by Tel-Aviv University Ethical Committee 27.3.18.

### Apparatus

#### EEG Device

Electroencephalography recordings were performed using the Neurosteer ^®^ single-channel EEG Recorder. A three-electrode medical-grade patch was placed on each subject’s forehead, using dry gel for optimal signal transduction. The non-invasive monopolar electrodes were located at the prefrontal regions; the difference between Fp1 and Fp2 in the International 10/20 electrode system produced the single-EEG-channel, with a reference electrode in Fpz, was ± 25 mV (Input noise < 30 nVrms); EEG electrode contact impedances were maintained below 12 kΩ, as measured by a portable impedance meter (EZM4A, Grass Instrument Co., West Warwick, RI, United States). The data were digitized in continuous recording mode at a 500-Hz sampling frequency. For further details, see Supplementary Appendix A.

A trained operator monitored each subject during recordings to minimize muscle artifacts and instructed each subject to avoid facial muscle movement during recordings, as well as alerted the subjects whenever they showed increased muscle or ocular movement. It should be noted that the differential input and the high common-mode rejection ratio (CMRR) assist in the removal of motion artifacts as well as line noise (Hoseini et al., 2021). The EEG power spectrum was obtained by fast Fourier transform (FFT) of the EEG signals within a 4-s window.

#### Signal Processing

The time-frequency approach to analyzing EEG data has been used in the past years to characterize brain behavior in AD (Jeong, 2004; Bibina et al., 2018; Nimmy John et al., 2019). Following this notion, we are using a novel time-frequency approach to analyze the EEG signal in this study. Full technical specifications regarding the signal analysis are provided in Supplementary Appendix A. In brief, the Neurosteer ^®^ signal-processing algorithm interprets the EEG data using a time/frequency wavelet-packet analysis, creating a presentation of 121 components composed of time-varying fundamental frequencies and their harmonics.

To demonstrate this process, let *g* and *h* be a set of biorthogonal quadrature filters created from the filters G and H, respectively. These are convolution-decimation operators where, in a simple Haar wavelet, *g* is a set of averages, and *h* is a set of differences.

Let ψ_1_ be the mother wavelet associated with the filters *s* ∈ *H*, and *d* ∈ *G*. Then, the collection of wavelet packets ψ_*n*_ is given by:


(1) $$\psi_{2n}=H\psi_{n};\psi_{2n}\left(t\right)=\sqrt{2}\sum_{j\in Z}\text{s}\left(j\right)\psi_{n}\left(2t-j\right),$$

(2) $$\psi_{2n+1}=G\psi_{n};\psi_{2n+1}\left(t\right)=\sqrt{2}\sum_{j\in Z}\text{d}\left(j\right)\psi_{n}\left(2t-j\right).$$

The recursive form provides a natural arrangement in the form of a binary tree. The functions _ψ_*n*__ have a fixed scale. A library of wavelet packets of any scale *s*, frequency *f*, and position *p* is given by


(3) $$\psi_{sfp}\left(t\right)=2^{-s/2}\psi_{f}\left(2^{-s}t-p\right).$$

The wavelet packets {ψ_*sfp*_:*p* ∈ *Z*} include a large collection of potential orthonormal bases. An optimal basis can be chosen by the best-basis algorithm (Coifman and Wickerhauser, 1992). Furthermore, an optimal mother wavelet together with an optimal basis can also be found (Neretti and Intrator, 2002). Following robust statistics methods to prune some of the basis functions using Coifman and Donoho’s denoising method (Coifman and Donoho, 1995), an output of 121 basis functions is received, termed “Brain Activity Features” (BAFs). Based on a given labeled-BAF dataset (collected by Neurosteer ^®^), various models can be created for different discriminations of these labels. In the (semi-) linear case, these models are of the form:


(4) $$V_{k}\left(w,x\right)=\text{Ψ}\left(\sum_{i}w_{i}x_{i}\right),$$

where *w* is a vector of weights and _Ψ_ is a transfer function that can either be linear, e.g., _Ψ_(*y*)=*y*__, or sigmoidal for logistic regression _Ψ_(*y*)=1/(1+*e*^–*y*^)__.

The BAFs are calculated over a 4-s window that is advanced by 1-s. This means that the BAF has 2048 components as it is a power of 2 and the sampling frequency is 500 Hz spanning the 4-s window. In this 4 s window, the BAF is a time/frequency atom. Thus, it allows for a signal that can vary the frequency over the 4-s window, such as a chirp. Then the window is advanced by 1 s, just like it is done in a spectrogram with 75% overlap, and calculated again over the new 4-s window.

The data was tested for artifacts due to muscle and eye movement of the prefrontal EEG signals (Fp1, Fp2). The standard methods used to remove non-EEG artifacts were all based on different variants of the Independent Components Analysis (ICA) algorithm (Urigüen and Garcia-Zapirain, 2015). These methods could not be performed here, as only a single-channel EEG data was used. As an alternative, strong muscle artifacts have higher amplitudes than regular EEG signals, mainly in the high frequencies; thus, they are clearly observable in many of the BAFs that are tuned to high frequency. This phenomenon helps in the identification of artifacts in the signal. Minor muscle activity is filtered out by the time/frequency nature of the BAFs and thus caused no disturbance to the processed signal. Similarly, eye movements are detected in specific BAFs and are taken into account during signal processing and data analysis.

#### Construction of Higher-Level Classifiers

Several linear combinations were obtained using ML techniques on labeled datasets previously collected by Neurosteer ^®^ using the described BAFs. Specifically, EEG features VC9 and A0 were calculated using the linear discriminant analysis (LDA) technique (Hastie et al., 2007). LDA technique is intended to find an optimal linear transformation that maximizes the class separability. LDA models on imaging data were found successful in predicting development of cognitive decline up to 4 years prior to displaying symptoms of decline (Rizk-Jackson et al., 2013). EEG feature VC9 was found to separate between low and high difficulty levels of an auditory detection task within healthy participants (ages 20–30). EEG feature A0 was found to separate between resting state with music and auditory detection task within healthy participants (ages 20–30).

EEG feature ST4 was calculated using PCA (Rokhlin et al., 2009). Principle component analysis is a method used for feature dimensionality reduction before classification. Studies show that features extracted using PCA show significant correlation to MMSE score and distinguish AD from healthy subjects (López et al., 2009; Meghdadi et al., 2021), as well as show good performance for the diagnosis of AD using imaging (Choi and Jin, 2018). Here, the fourth principal component was found to separate between low and high difficulty levels of auditory *n*-back task for healthy participants (ages 30–70). Most importantly, all three EEG features were derived from different datasets than the data analyzed in the present study. Therefore, the same weight matrices that were previously found were used to transform the data obtained in the present study.

The frequency approach has been extensively researched in the past decade, leading to a large body of evidence regarding the association of frequency bands to cognitive functions (Herrmann et al., 2016). In this study, we introduce a novel time-frequency approach for signal analysis and compare it to relevant frequency band results. The EEG features presented here are produced by a secondary layer of ML on top of the BAFs. These BAFs were created as an optimal orthogonal decomposition of time/frequency components following the application of the Best Basis Algorithm (Coifman and Wickerhauser, 1992) on the full wavelet packet tree that was created from a large collection of EEG recordings (see full details in the Supplementary Material). Therefore, they are composed of time-varying fundamental frequencies and their harmonics. As a result of this dynamic nature, and due to the fact that the EEG features are created as linear combinations of multiple BAFs, each feature potentially includes a wide range of frequencies and dynamic varying characteristics. If the time variant characteristic was not present, the spectral envelope of each feature would have represented the full characteristic of each EEG feature. As the time varying component of each BAF is in the millisecond range (sampled at 500 Hz), it is not possible to characterize the dynamics with a spectrogram representation which averages the signal over 4-s windows. Thus, though characterizing the frequency representations of the novel features may be of interest, it is not applicable in this case, much like with EEG-produced ERPs (Makeig et al., 2002; Fell et al., 2004; Popp et al., 2019). We do observe, however, that EEG feature VC9 includes predominantly fundamental frequencies that belong to the Delta and Theta range (and their harmonics), while EEG features ST4 and A0 are broader combinations of frequencies spanning the whole spectrum (up to 240 Hz).

Other studies conducted on young healthy participants (a different study population than that previously mentioned) showed that EEG feature VC9 activity increased with increasing levels of cognitive load, as manipulated by numeric *n*-back task (Maimon et al., 2020). Additionally, VC9 activity during the performance of an arithmetic task decreased with external visual interruptions (Bolton et al., 2021). VC9 activity was also found to decrease with the repetition of a motor task in a surgery simulator performed by medical interns and was correlated with their individual performance (Maimon et al., 2021). These studies found that VC9 showed higher sensitivity than Theta, especially for lower-difficulty cognitive loads, which are more suitable for clinical and elderly populations. Within the clinical population, VC9 was found to correlate with auditory mismatch negativity (MMN) ERP component of minimally responsive patients (Maimon et al., 2022). EEG feature ST4 was found to correlate to individual performance of the numeric *n*-back task. That is, the difference between high and low load in RTs per participant was correlated to the difference between high and low load in ST4 activity (Maimon et al., 2020).

### Procedure

#### EEG Recording and Auditory Battery

The recording room was quiet and illuminated. The research assistant set up the sanitized system equipment (electrode patch, sensor, EEG monitor, clicker) and provided general instructions to the participants before starting the task. Then the electrode was placed on the subject’s forehead, and the recording was initiated. Each participant was seated during the assessment and heard instructions through a loudspeaker connected to the EEG monitor. The entire recording session typically lasted 20–30 min. The cognitive assessment battery was pre-recorded and included two tasks: a detection task as well as a series of true/false questions answered by pressing a wireless clicker. Further explanations for the task were kept at a minimum to avoid bias. A few minutes of baseline activity were recorded per participant to ensure accurate testing. Each auditory cognitive assessment lasted 18 min.

##### Detection Task

**FIGURE 1:**
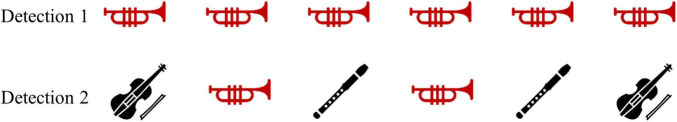
An example of six trials of detection level 1 **(Top)** and detection level 2 **(Bottom)**. Both examples show a “trumpet block” in which the participant reacts to the trumpet melody. Red icons represent trials in which the participant was required to respond with a click when hearing the melody, indicating a “yes” response.

Figure 1 illustrates the detection task used in the study. In each block, participants were presented with a sequence of melodies (played by a violin, a trumpet, and a flute). Each participant was given a clicker to respond to the stimuli. In the beginning of each block, auditory instructions indicated an instrument, to which the participant responded by clicking once. The click response was only to “yes” trials when the indicated instrument melody played. The task included two difficulty levels to test increasing cognitive load. In level 1, each melody was played for 3 s, and the same melody repeated throughout the entire block. The participant was asked to click once as fast as possible for each repetition of the melody. This level included three 90-s trials (one for each instrument), with 5–6 instances of each melody, and with 10–18 s of silence in between. In level 2, the same melodies were played for 1.5 s, and all three instruments appeared in the block. The participants were asked to click only for a specific instrument within the block and to ignore the rest of the melodies. Each trial consisted of 6-8 melodies, with 8–14 s of silence in between, and 2–3 instances of the target stimulus.

### Statistical Analysis

#### Dependent Variables

##### Behavioral Measurements

The behavioral dependent variables included mean response accuracy and mean Reaction times (RTs) per participant.

##### Electrophysiological (EEG) Variables

The electrophysiological dependent variables included the power spectral density. Absolute power values were converted to logarithm base 10 to produce values in dB. Out of the frequency bands, the following were included: Delta (0.5–4 Hz) and Theta (4–7 Hz). Pretests showed that the other frequency bands, namely Alpha (8–15 Hz), Beta (16–31 Hz), and lower Gamma (32–45 Hz), did not show any significant correlation or differences on the current data.

The analysis also included activity of the three selected EEG features: VC9, ST4, and A0, normalized to a scale of 0–100. The EEG variables were calculated each second from a moving window of 4 s, and mean activity per condition was factored into the analyses.

#### Overview

**FIGURE 2:**
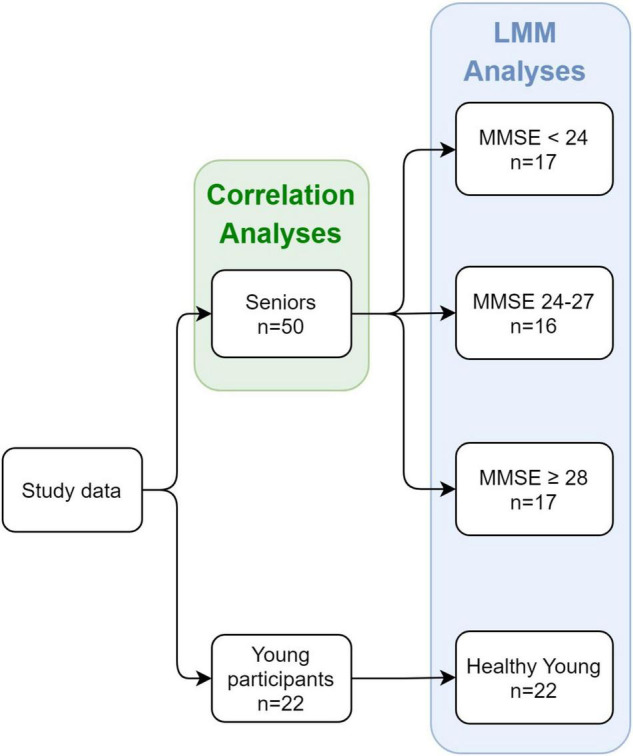
Study design and groups at each stage. The study included both seniors and young healthy participants as controls. For the senior participants, an MMSE score was obtained, and division into groups was based on the individual MMSE score.

Statistical analyses were performed on data from 50 senior participants and 22 healthy young participants (72 participants in total). Groups were allocated as follows (see Figure 2). The senior participants were divided into three groups according to their MMSE scores: (1) Patients with a score of 17–23 in the MMSE < 24 group (*n* = 17); (2) Patients with a score of 24–27 in the MMSE 24–27 group (*n* = 16); and (3) Patients with a score of 28–30 in the MMSE ≥ 28 group (*n* = 17). This was done in order to obtain relatively balanced group sizes. We used MMSE score cutoffs of 24 and 27 in allocating the groups, as we were mostly interested in detecting cognitive decline as early as possible and found previous indications that a higher cutoff score would achieve optimal evaluations of diagnostic accuracy (Crum et al., 1993). Furthermore, it was argued that educated individuals who score below 27 are at greater risk of being diagnosed with dementia (O’Bryant et al., 2008). The fourth group included in the analysis consisted of the 22 young healthy participants (healthy young group). To ensure that groups were well adjusted in terms of age and sex, we compared the mean ages of each MMSE group in total, and for males and females separately. Additionally, we compared the age and MMSE scores of each MMSE group between males and females. These comparisons were done with Welch Two Sample *t*-test. See Table 1 for the descriptive and statistical results.

The analyses included correlation models between EEG variables and MMSE score of senior participants, and mixed linear models measuring the associations between the EEG variables and MMSE score/group. Significance level for all analyses was set to *p* < 0.05. Post-hoc effects with Tukey corrections were made following significant main effect and interactions. All analyses were conducted with RStudio version 1.4.1717 (RStudio Team, 2020).

##### Correlation Analyses Between EEG Variables and MMSE Scores

**FIGURE 3:**
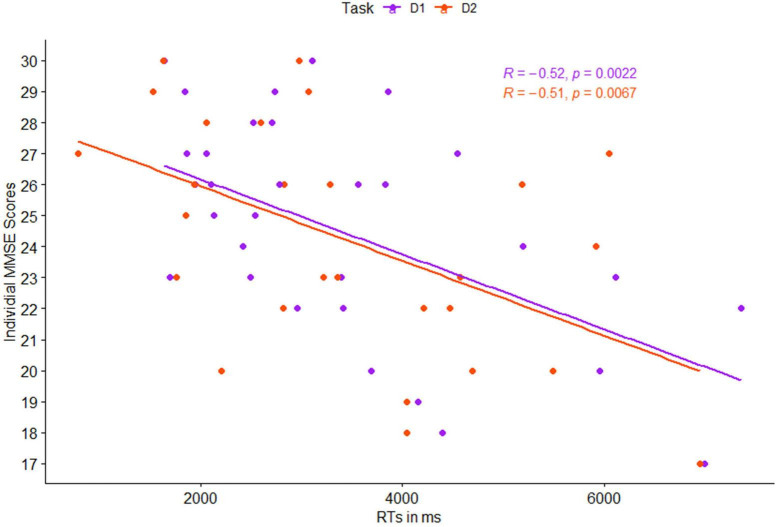
Pearson correlation between individual mean reaction times (RTs) and individual MMSE scores, as a function of task level: detection 1 (purple) and detection 2 (red).

As an initial validation of the cognitive assessment method and based on previous studies (Silverberg et al., 2011), we expected that the RTs in the cognitive detection task would be greater for participants with lower MMSE scores. This was tested by calculating the Pearson correlation coefficient between mean RTs in detection levels 1 and 2, and the individual MMSE score of each participant. Estimated correlation coefficient, corresponding 95% Confidence Interval (CI), and *p*-values are presented in Table 2 and Figure 3.

**TABLE 2:** Pearson *r* coefficients (first row of each cell), *p*-values (second row), and 95% CI (third row) of the correlations between individual MMSE scores and EEG features (and reaction times) as a function of task (averaged across tasks, resting state, detection level 1, and detection level 2); and the difference between correlation coefficients and CIs between tasks, as calculated by Meng et al. (1992) *z* extension of the Fisher Z transform (Dunn and Clark, 1969), including a test of the confidence interval for comparing two correlations.

	Averaged	Resting state	Detection level 1	Detection level 2	Resting state – Detection level 1	Resting state – Detection level 2	Detection level 1 – Detection level 2
RTs	*r* = –0.591, *p* = 0.0003**, (–0.78, –0.31)		*r* = –0.557, *p* = 0.0009***, (–0.76, –0.26)	*r* = –0.544, *p* = 0.003**, (–0.77, –0.21)			Δ*r* = –0.013, *p* = 0.92, (–0.37, 0.33)
Delta	*r* = 0.064, *p* = 0.656, (–0.22, –0.34)	*r* = 0.031, *p* = 0.8422, (–0.27, 0.33)	*r* = 0.086, *p* = 0.554, (–0.2, 0.36)	*r* = 0.044, *p* = 0.77, (–0.24, 0.33)	Δ*r* = –0.054, *p* = 0.67, (–0.31, 0.19)	Δ*r* = –0.012, *p* = 0.91, (–0.23, 0.2)	Δ*r* = 0.041, *p* = 0.55, (–0.1, 0.18)
Theta	*r* = 0.13, *p* = 0.367, (–0.15, –0.39)	*r* = 0.096, *p* = 0.541, (–0.21, 0.38)	*r* = 0.11, *p* = 0.426, (–0.17, 0.38)	*r* = 0.145, *p* = 0.329, (–0.15, 0.42)	Δ*r* = –0.02, *p* = 0.88, (–0.27, 0.24)	Δ*r* = –0.049, *p* = 0.68, (–0.29, 0.19)	Δ*r* = –0.031, *p* = 0.65, (–0.16, 0.1)
A0	*r* = –0.34, *p* = 0.016*, (–0.56, –0.07)	*r* = –0.245, *p* = 0.113, (–0.51, 0.06)	*r* = –0.38, *p* = 0.006**, (–0.6, –0.12)	*r* = –0.313, *p* = 0.033*, (–0.55, 0.03)	Δ*r* = 0.14, *p* = 0.047*, (0.001, 0.31)	Δ*r* = 0.068, *p* = 0.295, (–0.06, 0.21)	Δ*r* = –0.071, *p* = 0.24, (–0.2, 0.05)
ST4	*r* = 0.357, *p* = 0.011*, (0.09, 0.63)	*r* = 0.253, *p* = 0.102, (–0.05, 0.51)	*r* = 0.343, *p* = 0.015*, (0.07, 0.57)	*r* = 0.347, *p* = 0.017*, (0.07, 0.58)	Δ*r* = –0.09, *p* = 0.511, (–0.39, 0.2)	Δ*r* = –0.094, *p* = 0.357, (–0.33, 0.12)	Δ*r* = –0.005, *p* = 0.95, (–0.18, 0.17)
VC9	*r* = 0.076, *p* = 0.599, (–0.21, 0.35)	*r* = 0.067, *p* = 0.671, (–0.23, 0.36)	*r* = 0.094, *p* = 0.518, (–0.19, 0.36)	*r* = 0.071, *p* = 0.632, (–0.22, 0.35)	Δ*r* = –0.027, *p* = 0.79, (–0.23, 0.18)	Δ*r* = –0.01, *p* = 0.96, (–0.21, 0.2)	Δ*r* = 0.02, *p* = 0.69, (–0.08, 0.13)
| A0, MMSE| - | ST4, MMSE|	Δ*r* = –0.02, *p* = 0.89, (–0.32, 0.28)	Δ*r* = 0.09, *p* = 0.95, (–0.3, 0.28)	Δ*r* = 0.042, *p* = 0.77, (–0.28, 0.38)	Δ*r* = –0.034, *p* = 0.8, (–0.34, 0.27)			

Significant effects are presented in bold (*p < 0.05, **p < 0.01, ***p < 0.001).

**FIGURE 4:**
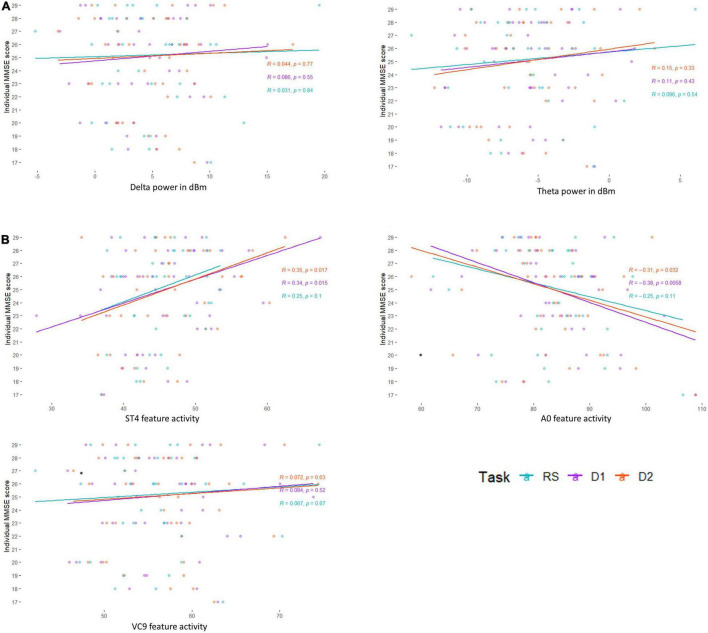
Pearson correlation between individual MMSE scores and **(A)** EEG frequency bands Delta and Theta, and **(B)** EEG features A0, ST4, and VC9, as a function of task level: resting state (blue), detection level 1 (purple), and detection level 2 (red).

Next, following our first hypothesis regarding the correlation between EEG activity and MMSE scores, we calculated the Pearson correlation between each of the EEG features and individual MMSE scores. Each feature’s activity was averaged across the three task conditions (i.e., detection level 1, detection level 2, and resting state), as well as averaged for each of the conditions separately. We then compared the Pearson correlations between the different tasks. This was done using Meng et al. (1992) *z* extension of the Fisher *Z* transform (Dunn and Clark, 1969), which includes a test of the confidence interval for comparing two correlations. We also compared significant correlations between the different features. All comparisons were done using the *Corcor* (Diedenhofen and Musch, 2015) library for Rstudio. Estimated correlation coefficients, 95% CIs, *p*-values, and their comparisons are summarized in Table 2 and visually presented in Figure 4.

Finally, we calculated partial Pearson product-moment partial correlations, controlling for age and built on the asymptotic confidence interval of the correlation coefficient based on Fisher’s *Z* transform, conducted with *RVAideMemoire* (Maxime, 2017) library in Rstudio. Each partial correlation between EEG variables and MMSE controlled for age was compared to the unadjusted correlation using the bootstrap method (Efron and Tibshirani, 1993). The bias-corrected and accelerated (BCa) bootstrap method was used to test if the difference between the overlapping adjusted, and unadjusted correlations is equal to zero. We used the *pzcor* function in *zeroEQpart* library with *k* = 1000 bootstrap samples taken, and *pzconf* function to calculate the subtraction of the partial correlation from the confidence intervals for the unadjusted correlation (Table 3).

**TABLE 3:** Partial Pearson *r* coefficients (first row of each cell), *p*-values (second row), and 95% CI (third row) of the correlations between individual MMSE scores and EEG features (and reaction times), controlled for age as a function of task (averaged across tasks, resting state, detection level 1, and detection level 2), and the difference between correlation coefficients and Cis, the partial correlations, and the unadjusted correlations, as calculated by the bias-corrected and accelerated (BCa) bootstrap method.

	Averaged	Resting state	Detection level 1	Detection level 2	Averaged	Resting state	Detection level 1	Detection level 2
	Partial correlation, age	Partial correlation, age	Partial correlation, age	Partial correlation, age	Unadjusted – partial correlation, age	Unadjusted – partial correlation, age	Unadjusted – partial correlation, age	Unadjusted – partial correlation, age
RTs	*r* = –0.531, *p* = 0.002**, (–0.75, –0.22)		*r* = –0.48, *p* = 0.006**, (–0.71, –0.15)	*r* = –0.522, *p* = 0.006**, (–0.76, –0.17)	Δ*r* = –0.06, *p* = 0.129, (–0.38, 0.07)		Δ*r* = –0.08, *p* = 0.19, (–0.48, 0.18)	Δ*r* = –0.02, *p* = 0.38, (–0.4, 0.06)
Delta	*r* = 0.004, *p* = 0.97, (–0.28, 0.29)	*r* = –0.041, *p* = 0.79, (–0.34, 0.27)	*r* = 0.021, *p* = 0.89, (–0.26, 0.3)	*r* < 0.001 *p* = 0.99, (–0.29, 0.29)	Δ*r* = 0.056, *p* = 0.09, (–0.082, 0.24)	Δ*r* = 0.073, *p* = 0.092, (–0.14, 0.32)	Δ*r* = 0.065, *p* = 0.075, (–0.06, –0.31)	Δ*r* = 0.044, *p* = 0.27, (–0.16, 0.25)
Theta	*r* = 0.07, *p* = 0.64, (–0.22, 0.34)	*r* = 0.047, *p* = 0.77, (–0.26, 0.35)	*r* = 0.048, *p* = 0.74, (–0.24, 0.33)	*r* = 0.08, *p* = 0.6, (–0.22, 0.36)	Δ*r* = 0.061, *p* = 0.07, (–0.11, 0.26)	Δ*r* = –0.049, *p* = 0.2, (–0.28, 0.25)	Δ*r* = 0.067, *p* = 0.1, (–0.1, 0.32)	Δ*r* = 0.066, *p* = 0.164, (–0.15, 0.41)
A0	*r* = –0.333, *p* = 0.0194*, (–0.56, –0.06)	*r* = –0.245, *p* = 0.113, (–0.51, 0.06)	*r* = –0.372, *p* = 0.008**, (–0.59, –0.1)	*r* = –0.303, *p* = 0.04*, (–0.55, –0.02)	Δ*r* = –0.01, *p* = 0.83, (–0.18, 0.24)	Δ*r* = –0.01, *p* = 0.84, (–0.28, 0.23)	Δ*r* = –0.01, *p* = 0.62, (–0.16, 0.18)	Δ*r* = –0.009, *p* = 0.78, (–0.2, – 0.23)
ST4	*r* = 0.321, *p* = 0.025*, (0.04, 0.55)	*r* = 0.266, *p* = 0.088, (–0.04, 0.53)	*r* = 0.291, *p* = 0.042*, (0.01, 0.53)	*r* = 0.293 *p* = 0.048*, (0.003, 0.54)	Δ*r* = 0.04, *p* = 0.25, (–0.14, 0.25)	Δ*r* = –0.013, *p* = 0.71, (–0.21, 0.41)	Δ*r* = 0.052, *p* = 0.128, (–0.09, 0.34)	Δ*r* = –0.054, *p* = 0.135, (–0.14, 0.32)
VC9	*r* = –0.004, *p* = 0.97, (–0.29, 0.28)	*r* = 0.007, *p* = 0.63, (–0.3, 0.31)	*r* = 0.002, *p* = 0.98, (–0.28, 0.28)	*r* = –0.01, *p* = 0.94, (–0.3, 0.28)	Δ*r* = 0.008, *p* = 0.051, (–0.055, 0.36)	Δ*r* = 0.06, *p* = 0.129, (–0.12, 0.28)	Δ*r* = 0.09, *p* = 0.072, (–0.12, 0.43)	Δ*r* = 0.082, *p* = 0.15, (–0.07, 0.39)
| A0, MMSE| - | ST4, MMSE|	*r* = 0.012, *p* = 0.93, (–0.29, 0.32)	*r* = –0.031, *p* = 0.82, (–0.32, 0.26)	*r* = 0.08, *p* = 0.58, (–0.24, 0.42)	*r* = 0.011, *p* = 0.94, (–0.29, 0.31)				

Significant effects are presented in bold (*p < 0.05, **p < 0.01, p < 0.001).

##### Associations Between EEG Variables and Groups

**FIGURE 5:**
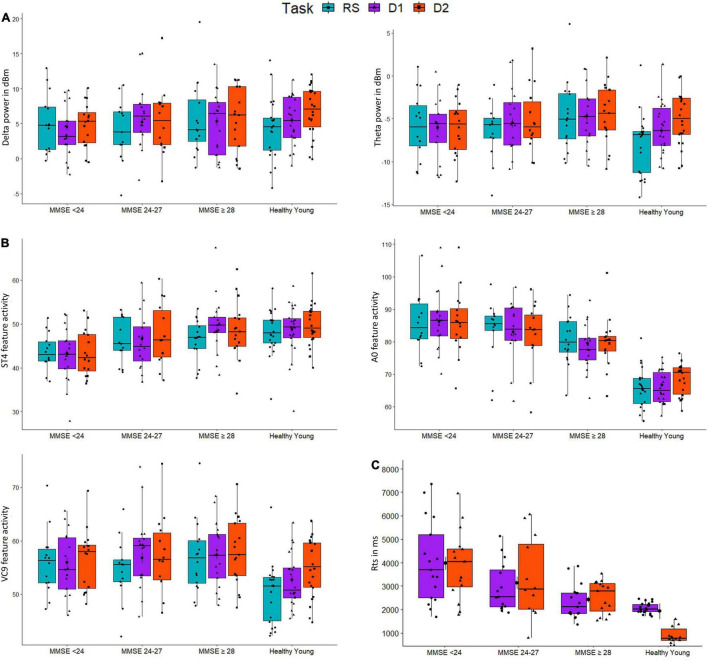
The distributions of groups (MMSE < 24, MMSE 24-27, MMSE ≥ 28, and healthy young) for activity of EEG features **(A)** Delta and Theta; **(B)** A0, ST4 and VC9; and **(C)** reaction times (RTs), as a function of task: resting-state (blue), detection level 1 (purple), and detection level 2 (red).

To create analyses detecting differences between groups, we added a group of young participants in addition to the three MMSE groups of the senior participants, all performing the same tasks. Due to the relatively small sample size, we fitted a general linear mixed model (GLMM) (Cnaan et al., 1997) that incorporated both fixed- and random-effect terms in a linear predictor expression from which the conditional mean of the response can be evaluated, using the *lmer* function in the *lme4* package (Bates et al., 2015). This model was chosen over the simple GLM due to the relatively small sample size, as the GLMM takes into consideration the random slope per each participant. Age was not inserted as a covariant since it was part of the analysis: the young healthy group is inherently different due to their ages. The model included the fixed within-participant variable of task level (resting state vs. detection level 1 vs. detection level 2), and group as between-participants variable (MMSE < 24 vs. MMSE 24–27 vs. MMSE ≥ 28 vs. healthy young). Both variables were coded as linear variables. Task: resting state = 0; detection level 1 = 1; and detection level 2 = 2. Group: MMSE < 24 = 0; MMSE 24–27 = 1; MMSE ≥ 28 = 2; healthy young = 3. The model included the samples per participant per task (i.e., samples per second of activation) as a random slope. The interaction between the two fixed variables and participants’ random slopes were fit to the EEG variable data in a step-wise-step-up procedure using Chi square tests: the initial model included group and task level without the interaction between them; the second model included both variables and the interactions, and afterwards, the random factor was fitted into the data to include task| participant as random slope (all model comparisons parameters are summarized in Table 4). Fixed effects were calculated according to the selected model (i.e., included the interaction between the variables only for selected models which included it), the fixed effects estimates, standard errors, DFs, *t* and *p*-values of the best-fitting models for each of the EEG variables are summarized in Table 5 and presented in Figure 5. For models that showed a significant main effect of either group or task, *post hoc* analyses were conducted, comparing possible pairwise comparisons of the main effects levels (i.e., comparing between groups and between task levels), using Tukey HDS correction. This was done using the *PostHocTest* function from the *DescTools* library in Rstudio. All pairwise differences, 95% Cis, and *p*-values are summarized in Table 6.

**TABLE 4:** Model type, number of parameters, AIC, BIC, loglik, deviance, Chi square, df and *p*-values of GLMM models selection for each of the EEG features.

	Interaction	Slope	npar	AIC	BIC	logLik	Deviance	Chi square	Df	*p*-value
Delta	Group + Task	1 | Participant	5	216883	216925	–108436	216873			
	Group × Task	1 | Participant	6	216827	216877	–108407	216815	58.2555	1	*<0.001*
	Group + Task	Task | Participant	7	215416	215475	–107701	215402	1412.7973	1	*<0.001*
	**Group × Task**	**Task | Participant**	**8**	**215412**	**215480**	–**107698**	**215396**	**6.0024**	**1**	***0.01429***
Theta	Group + Task	1 | Participant	5	201278	201320	–100634	201268			
	Group × Task	1 | Participant	6	201152	201203	–100570	201140	127.714	1	*<0.001*
	Group + Task	Task | Participant	7	199602	199661	–99794	199588	1552.228	1	*<0.001*
	**Group × Task**	**Task | Participant**	**8**	**199593**	**199661**	–**99789**	**199577**	**11.052**	**1**	***<0.001***
A0	Group + Task	1 | Participant	5	230037	230080	–115014	230027			
	Group × Task	1 | Participant	6	229961	230012	–114975	229949	77.8969	1	*<0.001*
	Group + Task	Task | Participant	7	227810	227869	–113898	227796	2153.9456	1	*<0.001*
	**Group × Task**	**Task | Participant**	**8**	**227807**	**227875**	–**113896**	**227791**	**4.1658**	**1**	***0.04125***
ST4	Group + Task	1 | Participant	5	253529	253572	–126760	253519			
	Group × Task	1 | Participant	6	253514	253565	–126751	253502	17.125	1	*<0.001*
	**Group + Task**	**Task | Participant**	**7**	**252581**	**252640**	–**126284**	**252567**	**934.999**	**1**	***<0.001***
	Group × Task	Task | Participant	8	252581	252648	–126282	252565	2.449	1	*0.1176*
VC9	Group + Task	1 | Participant	5	237457	237499	–118723	237447			
	Group × Task	1 | Participant	6	237365	237415	–118676	237353	94.156	1	*<0.001*
	Group + Task	Task | Participant	7	235879	235938	–117932	235865	1487.748	1	*<0.001*
	**Group × Task**	**Task | Participant**	**8**	**235872**	**235939**	–**117928**	**235856**	**9.196**	**1**	***0.002425***
RTs	Group + Task	1 | Participant	5	18829	18854	–9409.3	18819			
	Group × Task	1 | Participant	6	18830	18860	–9409.2	18818	0.3208	1	*0.5711216*
	**Group + Task**	**Task | Participant**	**7**	**18818**	**18853**	–**9402.2**	**18804**	**13.9718**	**1**	***0.0001856***
	Group × Task	Task | Participant	8	18820	18860	–9401.9	18804	0.5056	1	*0.4770534*

*The selected model is presented in bold.*

**TABLE 5:** Estimate, standard errors, df, *t*-values and *p*-values of the best fitted GLMMs conducted.

	Fixed Effect	Estimate	Standard error	Df	*t* value	Pr(> | t|)
Delta	Intercept	4.2818	0.7518	69.6859	5.695	<0.001
	Group	0.1402	0.4174	69.8016	0.336	0.737901
	Task	1.2123	0.313	72.1092	3.873	0.000234***
	Group × Task	–0.4384	0.1753	70.2362	–2.5	0.014739*
Theta	Intercept	–7.157	0.6306	70.3807	–11.349	<0.001
	Group	0.5531	0.3501	70.5204	1.58	0.118664
	Task	1.3308	0.2551	72.1439	5.216	<0.001***
	Group × Task	–0.4934	0.1429	70.3468	–3.452	<0.001***
A0	Intercept	67.9201	1.5947	71.413	42.591	<0.001
	Group	7.259	0.8853	71.6028	8.199	<0.001***
	Task	0.9746	0.4701	70.3273	2.073	0.0418*
	Group × Task	–0.5468	0.2639	69.2178	–2.072	0.042*
ST4	Intercept	48.5049	0.8781	69.7236	55.237	<0.001
	Group	–1.5885	0.4846	69.6139	–3.278	0.00163**
	Task	0.5254	0.3223	64.354	1.63	0.10795
VC9	Intercept	51.7001	1.1213	69.8791	46.107	<0.001
	Group	1.8839	0.6225	70.0068	3.026	0.00346**
	Task	2.3034	0.4276	71.5824	5.387	<0.001***
	Group × Task	–0.749	0.2395	69.8376	–3.127	0.00257**
RTs	Intercept	1866.45	265.55	93.85	7.029	<0.001
	Group	634.34	103.84	77.27	6.109	<0.001***
	Task	–11.86	157.68	33.08	–0.075	0.94

Significant effects are presented in bold (*p < 0.05, **p < 0.01, ***p < 0.001).

**TABLE 6:** Difference, 95% CI, and *p*-values of the *post hoc* comparisons for significant main effects of group and task.

	Effect	Contrast	5% CI	95% CI	*p*-value
Delta	Task	D1-RS	–1.2980921	2.103904	0.842
		D2-RS	–0.7001874	2.736718	0.343
		D2-D1	–1.0595261	2.290245	0.661
Theta	Task	D1-RS	–0.6079469	2.226128	0.370
		D2-RS	–0.185484	2.677673	0.102
		D2-D1	–0.9582807	1.832289	0.740
A0	Group	MMSE 24–27-MMSE < 24	–7.528808	1.1597942	0.232
		MMSE ≥ 28-MMSE < 24	–10.835796	–2.470839	<0.001***
		Healthy Young-MMSE < 24	–23.459068	–15.6007404	<0.001***
		MMSE ≥ 28-MMSE 24–27	–7.750391	0.8127688	0.157
		Healthy Young-MMSE 24–27	–20.379889	–12.3109055	<0.001***
		Healthy Young-MMSE ≥ 28	–16.736289	–9.016884	<0.001***
	Task	D1-RS	–3.251085	3.169276	1.000
		D2-RS	–2.729867	3.756377	0.926
		D2-D1	–2.606741	3.71506	0.910
ST4	Group	MMSE 24–27-MMSE < 24	0.1691464	6.382411	0.03438*
		MMSE ≥ 28-MMSE < 24	2.033485	8.01531	<0.001***
		Healthy Young-MMSE < 24	2.7283392	8.347871	<0.001***
		MMSE ≥ 28-MMSE 24–27	–1.3131616	4.810399	0.452
		Healthy Young-MMSE 24–27	–0.6227604	5.147413	0.180
		Healthy Young-MMSE ≥ 28	–2.2463862	3.273801	0.963
VC9	Group	MMSE 24–27-MMSE < 24	–2.483054	4.1764543	0.9123
		MMSE ≥ 28-MMSE < 24	–1.794501	4.616945	0.6648
		Healthy Young-MMSE < 24	–6.327589	–0.3044569	0.0246*
		MMSE ≥ 28-MMSE 24–27	–2.717159	3.8462025	0.9704
		Healthy Young-MMSE 24–27	–7.25502	–1.0704265	0.0033**
		Healthy Young-MMSE ≥ 28	–7.685571	–1.7689184	<0.001***
	Task	D1-RS	–0.9079706	4.013011	0.2978
		D2-RS	0.0875287	5.059007	0.0406*
		D2-D1	–1.4019719	3.443467	0.5808
RTs	Group	MMSE 24–27-MMSE < 24	–1872.9582	177.336	0.14217
		MMSE ≥ 28-MMSE < 24	–2534.7131	–574.4962	<0.001***
		Healthy Young-MMSE < 24	1099.0414	3003.1676	<0.001***
		MMSE ≥ 28-MMSE 24–27	–325.5364	1739.1236	0.28639
		Healthy Young-MMSE 24–27	197.5515	2209.0354	0.01207*

Significant effects are presented in bold (*p < 0.05, **p < 0.01, ***p < 0.001).

Finally, to explore whether the differences between task levels vary between the groups (hypothesis 4), we conducted separate GLMMs for each group, with task as a within-participants variable (coded as a linear variable similar to the main analysis). These included only the EEG features that exhibited significant interaction between group and task (i.e., only for features with selected model which included the interaction, and with significant interaction effect in their main GLMM). Taking all fixed effects together, we corrected the *p*-values using Benjamini Hochberg correction. For features that exhibited a corrected significant main effect of task using GLMM, we further compared the task levels using *post hoc* analyses with Tukey correction. The coefficients of the main effect of task using GLMMs are presented in Table 7, and post-hoc comparisons of significant features are presented in Table 8.

**TABLE 7:** Estimate, Standard errors, df, *t*-values and *p*-values, and BH-corrected *p*-values of the separate GLMM conducted on each group, with task level as a within-participants variable.

	Group	Fixed effect	Estimate	Standard error	Df	*t* value	Pr( > |t|)	Corrected *p* (BH)
Delta	Healthy Young	Task	1.5319	0.3662	22.4384	4.184	0.000372	0.002**
	MMSE ≥ 28	Task	0.1834	0.3498	16.6905	0.524	0.606995	0.883
	MMSE 24-27	Task	0.3956	0.4937	13.7184	0.801	0.436613	0.776
	MMSE < 24	Task	0.1172	0.3813	16.3312	0.307	0.762437	0.938
Theta	Healthy Young	Task	1.6119	0.3033	22.3316	5.314	2.36E-05	<0.001***
	MMSE ≥ 28	Task	0.218	0.3384	16.8459	0.644	0.528	0.845
	MMSE 24-27	Task	0.6692	0.2779	13.9808	2.408	0.0304	0.097
	MMSE < 24	Task	0.1831	0.6343	15.2962	0.289	0.777	0.888
A0	Healthy Young	Task	1.4866	0.5409	21.9732	2.748	0.0117	0.047*
	MMSE ≥ 28	Task	–0.1387	0.6072	17.1	–0.228	0.822	0.822
	MMSE 24-27	Task	–1.0817	0.5074	13.8666	–2.132	0.0514	0.137
	MMSE < 24	Task	0.1831	0.6343	15.2962	0.289	0.777	0.888
VC9	Healthy Young	Task	2.7553	0.5294	22.157	5.205	3.15E-05	<0.001***
	MMSE ≥ 28	Task	0.6391	0.5697	16.953	1.122	0.278	0.556
	MMSE 24-27	Task	1.1453	0.5479	13.5326	2.09	0.056	0.128
	MMSE < 24	Task	0.2415	0.4599	16.2038	0.525	0.607	0.809

Significant effects are presented in bold (*p < 0.05, **p < 0.01, ***p < 0.001).

**TABLE 8:** Estimate, SE, *t* and *p*-values of the *post hoc* comparisons for significant main effects of task in the sub GLMM conducted on healthy young participants.

	Contrast	Estimate	*SE*	*t* ratio	*p*-value (Bonferroni)
Delta Healthy Young	D1-D2	–1.41	0.684	–2.062	0.1362
	D1-RS	1.61	0.761	2.117	0.1206
	D2-RS	3.02	0.799	3.78	0.0015**
Theta Healthy Young	D1-D2	–1.03	0.583	–1.767	0.2534
	D1-RS	2.19	0.648	3.383	0.0047**
	D2-RS	3.22	0.681	4.732	0.0001***
A0 Healthy Young	D1-D2	–2.465	0.825	–2.988	0.014*
	D1-RS	0.698	0.917	0.762	1
	D2-RS	3.163	0.964	3.283	0.0062**
VC9 Healthy Young	D1-D2	–2.51	0.894	–2.809	0.0225*
	D1-RS	2.75	0.994	2.766	0.0252*
	D2-RS	5.26	1.044	5.036	<0.0001***

Significant effects are presented in bold (*p < 0.05, **p < 0.01, ***p < 0.001).

## Results

### Demographic Results

*T*-test results between the mean ages of each MMSE group in total, and for males and females separately as well as between the age and MMSE scores of each MMSE group between males and females were calculated. Means, standard deviations, *t* and *p*-values of the comparisons are presented in Table 1. The mean age of the participants did not differ between the three MMSE groups for all participants and for males and females separately (all *p*s > 0.05). Additionally, MMSE mean scores and mean age of each MMSE group were similar in males and females (all *p*s > 0.05).

### Validation of the Behavioral Task

Correlations between individual MMSE scores and participants’ RTs in both levels of the detection task were significant, both for each level separately, as well as the mean activation in the entire cognitive detection task (*p* < 0.01 for all, see Figure 3).

### Correlations Between MMSE Scores and EEG Variables

Pearson *r*, 95% CIs and *p*-values of the correlations between individual MMSE scores and each EEG variable (averaged and separated for each task level) and their comparisons are presented in Table 2 and Figure 4. The activity of ST4 increased with higher MMSE scores both as averaged across tasks, and separately during detection 2, and was highest during detection 1 (*p* = 0.011, *p* = 0.017, and *p* = 0.015, respectively). ST4 activity under the resting-state task did not correlate with MMSE (*p* = 0.102), and the comparison between the correlations during the different tasks did not yield significant differences. The activity of A0 increased with lower MMSE scores both as averaged across tasks and separately during detection 2, and was highest during detection 1 (*p* = 0.016, *p* = 0.033 and *p* = 0.006, respectively). A0 activity under the resting state task did not correlate with MMSE and was significantly lower than the correlation during detection level 1 (*p* = 0.113 and *p* = 0.047, respectively). There was no difference between the correlations of ST4 and MMSE and A0 and MMSE. No other features were found to be correlated with individual MMSE scores.

The partial Pearson correlations between the EEG features and MMSE scores controlled for age demonstrated fully comparable results to the unadjusted correlations: A0 and ST4 showed significant partial correlations with MMSE both for the averaged activity and separately during detection level 1 and detection level 2. All partial correlations controlling for age were not significantly different from the unadjusted correlation, and partial correlations between MMSE and A0 were no different from the partial correlations between MMSE and ST4 (see Table 3).

### Associations Between EEG Variables and Groups

For EEG features Delta, Theta, A0, and VC9, the best fitted model included the fixed effects of group, task, and the interaction between them; and the random slope included task/participant. For ST4 and reaction times, the best fitted model included fixed effects of group and task without their interaction, and the random slope task| participant (see Table 4 for model selection and Chi test results).

The distribution of participants’ EEG features activity under the three tasks is presented in Figure 5. All the model fixed effects estimates, standard errors, DFs, and *t* and *p*-values are presented in Table 5. The main linear effect of group was significant for EEG features ST4, A0, and VC9 (*p* < 0.001, *p* < 0.001, and *p* = 0.003, respectively), and for RTs (*p* < 0.001). *Post hoc* comparisons using Tukey corrections revealed that for EEG feature A0, significant differences were found between young healthy participants and all the senior groups, as well as the difference between the senior group with MMSE ≥ 28 and the senior group with MMSE < 24 (all *p*s < 0.001). EEG feature ST4 differentiated between the senior group with MMSE < 24 and the senior group with MMSE between 24 and 27, the senior group with MMSE ≥ 28 and the healthy young participants (*p* = 0.034, *p* < 0.001, and *p* < 0.001, for the comparisons between senior group with MMSE < 24 and MMSE 24–27, MMSE ≥ 28 and healthy young participants, respectively). EEG feature VC9 showed significant comparisons between the young healthy group and all senior groups (*p* = 0.024, *p* = 0.003, and *p* < 0.001 for the comparisons between healthy young group and senior group MMSE < 24, MMSE 24–27, and MMSE ≥ 28, respectively). Finally, RT results showed significant comparisons between the healthy young participants and the senior groups with MMSE < 24, and MMSE between 24 and 27 (*p* < 0.001 and *p* = 0.012, respectively), as well as between senior group with MMSE ≥ 28 and the senior group with MMSE < 24 (*p* < 0.001). For all significant pairwise comparisons results, see Table 6.

The main effect of task was significant for Delta, Theta, A0, and VC9 (*p* < 0.001, *p* < 0.001, *p* = 0.042, and *p* < 0.00, respectively). However, *post hoc* comparisons of task main effect levels revealed that the difference between detection level 2 and resting state was significant only for VC9 (*p* = 0.041).

Finally, the interaction between group and task level was significant for Delta, Theta, A0 and VC9 (*p* = 0.015, *p* < 0.001, *p* = 0.042, and *p* = 0.003, respectively). To unfold this interaction for each variable separately, we conducted GLMMs per each group with task as a within-participants variable. Results revealed that for all the variables (after BH correction) the main linear effect of task was significant only for the healthy young participants group (*p* = 0.002, *p* < 0.001, *p* = 0.047, and *p* < 0.001 for Delta, Theta, A0 and VC9, respectively). See Table 7 for the separate GLMMs per each group. Post-hoc pairwise comparisons with Bonferroni correction revealed that in the young healthy group, the difference between detection level 2 and resting state was significant for all features (*p* = 0.002, *p* < 0.001, *p* = 0.006, and *p* < 0.001 for Delta, Theta, A0 and VC9, respectively). The difference between detection level 1 and resting state was significant only for Theta and VC9 (*p* = 0.004, and *p* = 0.025, respectively). The difference between the detection levels 1 and 2 was significant for A0 and VC9 (*p* = 0.014, and *p* = 0.025, respectively). See Table 8 for all pairwise comparisons.

## Discussion

Cognitive decline remains highly underdiagnosed (Lang et al., 2017). Improving the detection rate in the community to allow early intervention is therefore imperative. The aim of this study was to evaluate the ability of a single-channel EEG system with an interactive assessment tool to detect cognitive decline with correlation to known assessment methods. We demonstrate that objective EEG features extracted from a wearable EEG system with an easy setup, together with a short evaluation, may provide an assessment method for cognitive state.

Fifty seniors and twenty-two healthy young control participants completed a short auditory cognitive assessment battery. Classical EEG frequency bands as well as pre-defined ML features were used in the analysis of the data. ML applied to EEG signals is increasingly being examined for detection of cognitive deterioration. The biomarkers that are extracted using ML approaches show accurate separation between healthy and cognitively impaired populations (Cichocki et al., 2005; Melissant et al., 2005; Amezquita-Sanchez et al., 2019; Schapkin et al., 2020; Doan et al., 2021; Meghdadi et al., 2021). Our approach utilizes wavelet-packet analysis (Coifman and Wickerhauser, 1992; Neretti and Intrator, 2002; Intrator, 2018; Intrator, 2019) as pre-processing to ML. The EEG features used here were calculated using a different dataset to avoid the risks associated with classification studies, such as overfitting (Mateos-Pérez et al., 2018). This is unlike other studies that use classifiers trained and tested via cross validation on the same dataset (Deiber et al., 2015; Kashefpoor et al., 2016; Khatun et al., 2019). Specifically, the pre-extracted EEG features used here, VC9 and ST4, were previously validated further in studies performed on healthy young subjects. Results showed a correlation of VC9 to working memory load (Maimon et al., 2020, 2021; Bolton et al., 2021) and a correlation of ST4 to individual performance (Maimon et al., 2020).

The wearable single-channel EEG system was previously used in several studies to assess cognition (Maimon et al., 2020, 2021, 2022; Bolton et al., 2021). A novel cognitive assessment based on auditory stimuli with three cognitive load levels (high, low, and rest) was used to probe different cognitive states. Individual response performance (RT) was correlated to the MMSE score in both difficulty levels of the cognitive task, which further validates the cognitive assessment tool. The auditory stimuli included a simple detection task involving musical stimuli. We chose this particular detection task because it is one of the most commonly used tasks to measure differences in EEG activity between cognitive decline groups (Paitel et al., 2021), and it requires relatively low cognitive load levels, which is well suited for cognitive decline states (Debener et al., 2005). We used this cognitive assessment on senior participants in different cognitive states (from healthy seniors to cognitive decline patients, as determined by MMSE score independently obtained by clinicians), and on healthy young participants.

Verifying our first hypothesis, activity of EEG features A0 and ST4 and RTs significantly correlated with individual MMSE scores for both levels of the auditory detection task. These correlations persisted when controlling for age, thus eliminating a possible confounding effect. Additionally, the correlations between MMSE scores and EEG features ST4 and A0 activity were significant for both difficulty levels of the cognitive task (i.e., detection levels 1 and 2). Comparison of the correlations showed that the low difficulty level of a detection task elicited the highest correlation to MMSE scores, specifically for A0. These correlation analyses indicate a significant initial association between the novel EEG features and cognitive states as previously determined by clinical screening tools.

To continue exploring this association, further analysis compared the senior groups with the addition of a control group of healthy young participants. Results demonstrated the ability of the EEG features A0 and ST4, as well as RTs, to significantly differentiate between groups of seniors with high vs. low MMSE scores. High MMSE scores are associated with healthy cognition, while low MMSE scores tend to indicate a cognitive impairment. In allocating the groups, we used the common cutoff score of 24 to divide between low-functioning (MMSE < 24) and high-functioning seniors. However, we divided the high-functioning group further using a cutoff score of 27 to get a notion of possible separability between cognitive states in high-functioning seniors. Results showed that EEG features ST4 and A0 separated between the group of seniors with high-MMSE scores and the low-MMSE group, with the common cutoff score of 24, comparable to previous reports in the field (Lehmann et al., 2007; Kashefpoor et al., 2016; Khatun et al., 2019). Additionally, ST4 showed differences between the low MMSE group (MMSE < 24) and the young healthy group, which is expected, but it also showed a significant difference to the group of seniors with MMSE 24–27 scores. Finally, although RTs exhibited some significant differences between the groups (i.e., between the healthy young participants and the senior groups with MMSE 24–27 and MMSE < 24, and between the group with MMSE ≥ 28 and MMSE < 24), EEG features ST4 and A0 show additional more subtle differences between the groups that were not detectable using behavioral performance alone (e.g., the difference in ST4 activity between MMSE < 24 and MMSE 24–27).

These results suggest a detection of more delicate differences between seniors with MMSE scores under 24 and seniors that are considered healthy to date, but are at a greater risk for developing cognitive decline (with MMSE scores below 27 but above 24). The results may further indicate a different cognitive functionality between seniors that are already considered to have experienced a certain decline (MMSE < 24) and seniors that score lower in the initial screening test but are not considered as suffering from cognitive decline (MMSE 24–27). This result contributes to the debate in the literature over cognitive functionality of patients with scores below 27 (Shiroky et al., 2007; O’Bryant et al., 2008). Finally, EEG features A0 and VC9 showed significant differences between the young healthy group and all of the senior groups. While a separation between healthy controls and low-MMSE score groups is expected, these results also suggest different cognitive patterns between healthy young participants and seniors considered healthy (based on their MMSE scores), consistent with reports from previous studies (Vlahou et al., 2014).

Confirming our third hypothesis that EEG activity will correlate with cognitive load levels, results further demonstrated that the task variable modulated EEG features A0, VC9 and Theta activity, and was correlative to cognitive load level. Activity of these features increased with higher cognitive load only within healthy young group and not in the senior groups, who did not exhibit such activity patterns, corroborating our fourth hypothesis. Although all features exhibited a significant difference between the two cognitive load extremes, only VC9 feature showed significant effects for all of the comparisons between the different levels of cognitive load. This is in line with previous reports of frontal Theta showing an increase during cognitively demanding tasks (Jensen and Tesche, 2002; Scheeringa et al., 2009). This difference was not present in the senior population, supporting the notion that Theta may be indicative of cognitive state and serve as a predictor of cognitive decline, consistent with previous findings (Deiber et al., 2015; Missonnier et al., 2007). Results of VC9 activity are also consistent with previous work, supporting the association of VC9 with working memory load in the healthy population (Maimon et al., 2020, 2021; Bolton et al., 2021). All together, these results provide an initial indication of the ability of the proposed tool to assess cognitive states and detect cognitive decline in the elderly population.

Taking these new results together with previous reports, EEG feature VC9 shows clear association to frontal brain functions involving cognitive load, closely related to frontal Theta (Maimon et al., 2020, 2021, 2022; Bolton et al., 2021). Further, EEG feature ST4, previously shown to correlate with individual performance of healthy young participants undergoing highly demanding cognitive load task (n-back task with 3 levels; Maimon et al., 2020), was found in the present study to correlate to MMSE score of seniors in different states of cognition, showing specific sensitivity for lower MMSE scores. As such, this feature may be related to general cognitive abilities, and specifically most sensitive to declining cognitive state. Finally, EEG feature A0 exhibited a correlation to MMSE score within senior participants, as well as ability to differentiate between groups of cognitive decline and healthy participants, with higher sensitivity to the higher levels of cognitive state (i.e., the healthy young participants and senior participants with no cognitive decline). In addition, it also correlated with cognitive load within the healthy young participants. Therefore, we concur that A0 might be related to brain functions involving cognitive load and abilities within the cognitively healthy population and can detect gentle changes of brain activity in the slightly impaired population.

The ability to differentiate between cognitive states with the EEG features shown here relies solely on a single EEG channel together with a short auditory assessment, unlike most studies attempting to assess cognitive states with multichannel EEG systems (Dauwels et al., 2010b; Moretti et al., 2011). It has been argued that the long setup time of multichannel EEG systems may cause fatigue, stress, or even change mental states, affecting EEG patterns and, subsequently, study outcomes (Cassani et al., 2017). This suggests that cognitive state evaluation using a wearable single-channel EEG with a quick setup time may not only make the assessment more affordable and accessible, but also potentially reduce the effects of pretest time on the results. Using a single EEG channel was previously shown to be effective in detection of cognitive decline (Khatun et al., 2019); however, here we demonstrate results obtained using features that were extracted from an independent dataset to avoid overfitting the data. The assessment method offered here may potentially enable detection of cognitive decline in earlier stages, before major dementia symptoms arise.

While this pilot study shows promising initial results, more work is needed. Specifically, additional studies should include a longer testing period to quantify the variability within subjects and to potentially increase the predictive power. Due to the small sample size, generalization of the results is limited. Thus, larger cohorts of patients that are quantified by extensive screening methods would offer an opportunity to get more sensitive separation between earlier stages of cognitive decline using the suggested tool, and potentially reduce the subjective nature of the MMSE. Furthermore, thorough diagnostic batteries such as the Petersen criteria (Petersen, 2004), Clinical Dementia Rating (Morris, 1993), and NINCDS-ADRDA (Albert et al., 2011) would assist in determining the patient’s clinical stage (i.e., MCI/dementia) and may provide further diagnostic predictions in addition to screening. Moreover, a longitudinal study could assess cognitive state in asymptomatic senior patients and follow participants’ cognition over an extended period of time, validating the predictive power of the EEG features. Education levels of the senior participants were not collected in the present study, presenting a key limitation. Education level was previously shown to effect individual MMSE scores (Crum et al., 1993) and including such data could improve the models if taken as a covariate in the statistical methods (Choi et al., 2019). Further studies with the novel EEG features should include education level data and explore their correspondences to MMSE scores. Finally, our approach utilizes wavelet-packet analysis as pre-processing to ML, creating components composed of time-varying fundamental frequencies and their harmonics. As a result, analyzing the features only in terms of frequency range takes away two important properties of these components: their fine-temporal nature and their reliance on harmonics of the fundamental frequency. The best analogy to this can be found in the visual cortex; simple cells in the visual cortex respond to bars at a certain orientation while complex cells respond to a collection of moving bars at different orientations and velocity, which are formed from collections of simple cells (Hubel and Wiesel, 1962), rendering the complex cells crucial to representation of 3D structure (Edelman and Intrator, 2000). Our approach is similar, where the complex time/frequency components of this dynamic nature are instrumental in the interpretation of the EEG signal. Future research should explore the usefulness of this approach in cognitive assessment. Furthermore, exploring the potential usefulness of the novel EEG features presented here in controlled studies characterizing EEG psychogeography in seniors may contribute to understanding the association of these features to basic brain function.

## Conclusion

This pilot study successfully demonstrated the ability to assess cognitive states using a wearable single-channel EEG and machine-learning EEG features that correlate to well-validated clinical measurements for detection of cognitive decline. Using such a low-cost approach to allow objective assessment may provide consistency in assessment across patients and between medical facilities clear of tester bias. Furthermore, due to a short setup time and the short cognitive evaluation, this tool has the potential to be used on a large scale in clinics in the community to detect deterioration before clinical symptoms emerge. Future studies should explore potential usefulness of this tool in characterizing changes in EEG patterns of cognitive decline over time, for early detection of cognitive decline to potentially allow earlier intervention.

## Data Availability Statement

The datasets presented in this article are not readily available because the datasets generated and analyzed during the current study are not publicly available due to patient privacy but may be available from the corresponding author under restrictions. Requests to access the datasets should be directed to NI, nathan@neurosteer.com.

## Ethics Statement

Ethical approval for this study was granted by the Ethics Committee of Dorot Geriatric Medical Center (for seniors) and Tel Aviv University Ethics Committee (for young healthy participants). Registration: Israeli Ministry of Health (MOH) registry number MOH_2019-10-07_007352. NIH Clinical Trials Registry number NCT04386902.

## Author Contributions

LM, NM, NI, and AS: conception and study design. LM, NR-P, and SR: data acquisition. NI and AS: supervision. LM, NM, and NI: data analysis and writing. All authors read and approved the final manuscript.

## Conflict of Interest

LM, NM, and NI have equity interest in Neurosteer, which developed the Neurosteer system. The remaining authors declare that the research was conducted in the absence of any commercial or financial relationships that could be construed as a potential conflict of interest.

## Publisher’s Note

All claims expressed in this article are solely those of the authors and do not necessarily represent those of their affiliated organizations, or those of the publisher, the editors and the reviewers. Any product that may be evaluated in this article, or claim that may be made by its manufacturer, is not guaranteed or endorsed by the publisher.
